# Coolidge effect in pond snails: male motivation in a simultaneous hermaphrodite

**DOI:** 10.1186/1471-2148-7-212

**Published:** 2007-11-06

**Authors:** Joris M Koene, Andries Ter Maat

**Affiliations:** 1 Department of Animal Ecology, Faculty of Earth & Life Sciences, Vrije Universiteit, De Boelelaan 1085, 1081 HV Amsterdam, The Netherlands; 2 Max-Planck-Institut für Ornithologie, Postfach 1564, 82305 Starnberg, Germany

## Abstract

**Background:**

The simultaneously hermaphroditic pond snail, *Lymnaea stagnalis*, can mate in the male and female role, but within one copulation only one sexual role is performed at a time. Previous work has shown that male motivation is determined by the availability of seminal fluid in the prostate gland, which is detected via a nervous connection by the brain area controlling male behaviour. Based on this knowledge, patterns of sexual role alternations within mating pairs can be explained.

**Results:**

The data presented here reveal that these snails can donate and receive sperm several times within 24 hours, and that they have increased mating rates in larger groups (i.e. more mating opportunities). For mating pairs we show, by introducing novel mating partners after copulation, that animals do inseminate new partners, while they are no longer motivated to inseminate their original partners.

**Conclusion:**

Our findings provide the first direct evidence for higher motivation in a hermaphrodite to copulate when a new partner is encountered. This Coolidge effect seems to be attenuated when mucus trails are excluded, which suggests that a chemical or textural cue may be responsible for mediating this response to sperm competition.

## Background

Males of many species distribute their sperm over several mates, and often do this in a way that is theoretically predicted to maximize their lifetime reproductive success [e.g., [1,2]]. Since the seminal paper by Dewsbury [3], it is now generally accepted that sperm and seminal fluids are costly to produce and that males are selected to strategically allocate their sperm [4]. One behavioural strategy to achieve the latter is via rekindled sexual motivation upon encounter of a new partner, which has been dubbed the Coolidge effect by Whalen in 1959 (after an anecdote about President and Mrs. Coolidge) [5]. This phenomenon of preferentially inseminating a novel partner was first demonstrated experimentally in rats [6], and is generally assumed to be widespread among promiscuous vertebrates [4,5,7]. The Coolidge effect is based on familiarity with a mating partner, and is often assumed to require the presence of an individual recognition mechanism. However, individual recognition is not included in the definition and has, to our knowledge, actually never been shown to lay at the basis of this phenomenon. Nonetheless, the Coolidge effect is usually thought to require cognitive brain areas involved in learning and memory that can integrate the information from different neural systems involved in individual discrimination, novelty detection and memory maintenance, and motivation [e.g., [7,8]].

Darwin [9] stated about molluscs, and other species belonging to the "lower classes", that "it is almost certain that these animals have too imperfect senses and much too low mental powers, to appreciate each other's beauty or other attractions, or to feel rivalry". However, it is now clear that sexual selection and sperm competition are widespread among invertebrates [e.g., [10]]. Here, we show that the Coolidge effect is also not restricted to vertebrates, because we find that this motivational strategy is used by a hermaphroditic invertebrate.

There is now abundant evidence that insects are prudent with their expensive sperm and seminal fluids, despite their relatively high mating rates. Gage [11,12] was the first to show that for the medfly, *Ceratitis capitata*, the presence of sperm competition results in the transfer of more sperm per ejaculate. Subsequent studies further focused on sperm numbers as a response to the risk or intensity of sperm competition [e.g., [13-18]]. Interestingly, we have only been able to find one insect study, on *Tribolium castaneum*, that specifically looked at whether males prefer to inseminate novel females over familiar females, which they had already inseminated [19]. Although that study provided evidence for a Coolidge effect this connection was somehow not made at the time, possibly because of the lack of knowledge about the underlying motivational mechanism. Another study, on *Gryllus bimaculatus*, showed that females preferred to mate with a novel male, indicating that the Coolidge effect is not limited to males [20].

The great pond snail, *L. stagnalis*, is a simultaneous hermaphrodite that can mate in the male and female role, but within a single copulation one sexual role is performed (and roles can be swapped afterwards) [21]. These snails seem usually prepared to receive sperm [21], they can receive sperm multiple times within a breeding season [22], and they can store and use the received sperm from different partners [23]. However, they will only donate sperm after several days of sexual isolation [24]. Previous studies have demonstrated that this male motivation is determined by the amount of seminal fluid in the prostate gland [25]. The gland's volume increase is detected, via a nervous connection, by an evolutionarily conserved brain region controlling male behaviour in gastropod molluscs [26].

*L. stagnalis*' male behaviour comprises a fixed sequence of events. These events include shell mounting and circular locomotion over the partner's shell to eventually find the position on the edge of the shell from where the female opening is accessible. This is followed by probing with the everted preputium (penis carrying organ) and, once the female gonopore is found, penis intromission and transfer of a copious amount of semen [24]. That the male reproductive investment of this simultaneous hermaphrodite equals the energetic costs of female reproduction was demonstrated by experimentally eliminating male behaviour, which resulted in a doubling of egg production [27]. The above illustrates that these snails are expected to be prudent with their expensive male reserves. It would be prudent to use the sperm to inseminate different partners rather than the same partner several times, which we set out to test here.

## Results

**Figure 1:**
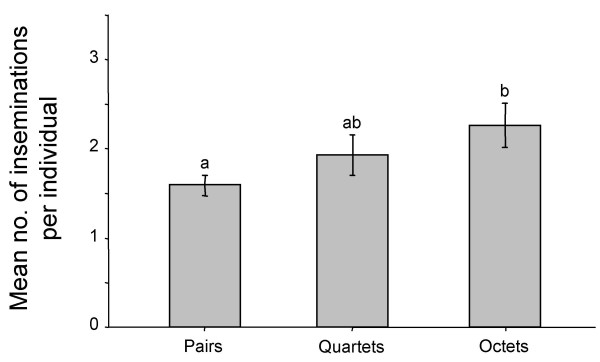
24 hour observations of copulation activity in groups of 2, 4 and 8 snails. The mean number of inseminations per individual are shown, the error bars represent standard errors of the mean of the replicates. For clarity the replicates are pooled. Groups with different letters (a or b) differ significantly from each other.

The mean number of inseminations per individual observed during 24 hour observations of Pairs, Quartets, and Octets are shown in Figure 1. The overall difference between the groups was significant (Nested ANOVA: *F*_2,18 _= 3.88, *p *= 0.027). Post-hoc testing showed that Octets had a higher insemination frequency than Pairs (Tukey HSD, *p *< 0.05), while the Quartets' frequency was in between but different from neither.

**Figure 2:**
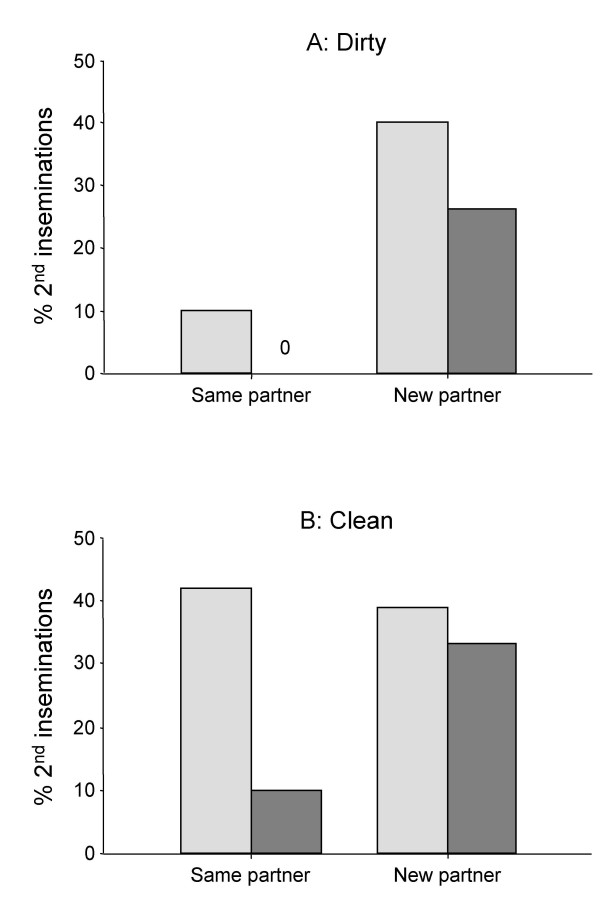
Copulations after the exchange of partners. The percentages of animals inseminating their partner during the second 6 hours (i.e. performing a second insemination) are shown for the dirty aquarium (**A**) and the clean aquarium (**B**). The different shades of grey indicate the two replicates.

In pairs of snails we subsequently tested experimentally whether the observed increase in insemination frequency in larger groups was due to the presence of new partners. We used a mixed model (nominal logistic REML procedure) to test for the fixed effects of treatment (New/Same partner) and test (Dirty/Clean aquarium) and the random effect of replicates (nested in treatment) on whether insemination occurred. Prior to the treatment, i.e. during the first 6 hours of the experiment, on average 39.25 (± 1.71 S.D., N = 4 replicates) of the 48 animals inseminated their partner. For this observation period no differences were found in the number of inseminations (treatment: χ^2 ^< 0.00, *df *= 1, *p *= 1.00; test: χ^2 ^= 0.31, *df *= 1, *p *= 0.57; interaction: χ^2 ^= 0.07, *df *= 2, *p *= 0.96). In all cases where insemination took place after the treatment, i.e. during the second 6 hours, animals inseminated their partners only once. Significantly more new partners were inseminated than familiar partners (χ^2 ^= 6.14, *df *= 1, *p *= 0.013). In a clean aquarium more copulations were observed than in an uncleaned aquarium (χ^2 ^= 3.88, *df *= 1, *p *= 0.049), indicating that the presence of mucus is important for this effect. The interaction between the two factors was also significant (χ^2 ^= 6.84, *df *= 2, *p *= 0.033), suggesting that the effect of a new partner depended on the factor Clean/Dirty. The percentage of animals performing an insemination during the second 6 hours is shown separately for the two replicates in the Dirty (Figure 2A) and the two replicates in the Clean aquaria (Figure 2B).

Insemination durations were not significantly different between the first and second 6 hours of the tests with the Dirty aquaria (Paired *t*-test: Replicate 1, Same partner, *t *= 0.143, *df *= 1, *p *= 0.910; New partner, *t *= 0.252, *df *= 7, *p *= 0.808; Replicate 2, New, *t *= -1.227, *df *= 4, *p *= 0.287; Same not tested because none mated in second 6 hours). Neither were they different in the tests with the Clean aquaria (Replicate 1, Same, *t *= -0.153, *df *= 7, *p *= 0.883, New, *t *= -2.282, *df *= 7, *p *= 0.056; Replicate 2, Same, *t *= -3.75, *df *= 1, *p *= 0.166, New, *t *= -2.408, *df *= 6, *p *= 0.053). For the latter however, when a new partner was present, in both cases the trend was that insemination duration was slightly shorter in the second 6 hours.

## Discussion

Dewsbury [4] predicted that in many species performing the male role will be a costly event. The high costs of the male function have previously been demonstrated in the simultaneously hermaphroditic pond snail *L. stagnalis *[27]. Hence, these snails can be expected to be prudent with their expensive male reserves (sperm and seminal fluid). We here suggest that they achieve a strategic allocation of their sperm by preferentially inseminating different partners, possibly via an inhibition of inseminating the same partner twice. The existence of such a preference is supported by our observation that the number of inseminations per individual increases with group size. More importantly, we show experimentally that the motivation to mate as a male is lower when the same partner is encountered than when a novel partner is encountered.

Although *L. stagnalis *is not likely to remate with its original partner after having inseminated its partner once [25], we now find that this species readily inseminates a new partner. Clearly, this motivational strategy helps to assure distribution of sperm over several partners and prevents depletion of the autosperm store on a single mate. Although previously suggested for *Aplysia fasciata *[28], the current study provides the first direct evidence for a Coolidge effect, defined as a higher motivation to copulate when a new partner is encountered, in a simultaneous hermaphrodite. This finding is consistent with the higher number of inseminations per individual in larger groups and provides the first experimental evidence for the efficient distribution of copulations in a simultaneous hermaphrodite. Moreover, it supports the male-biased mating decisions predicted by the "gender ratio hypothesis" that was recently proposed for simultaneous hermaphrodites [29].

Mature *L. stagnalis *are usually receptive for sperm and recipients remain relatively inactive during mating [24]. Hence, it seems that it is the level of male sexual drive that determines total copulatory activity [25]. Sperm donors, moreover, court actively by mounting the partners' shell and perform a series of characteristic behaviours. This leads us to conclude that the introduction of a new partner increases the hermaphrodite's male motivation. We therefore propose that the model for male sexual drive in molluscs, which is currently mainly based on prostate gland size [25], needs to be extended. Our results indicate that, besides the availability of sufficient seminal fluid, the number of available mating opportunities also influences the motivation to mate in the male role. It should be noted that we did not look at possible insemination avoidance or facilitation by the recipient. If such behaviours exist in this species, they would clearly also affect the occurrence of insemination.

The results differ when a dirty or clean aquarium is used. The fact that the effect of a new partner is attenuated in a novel environment, combined with the finding that even with the same partner, the number of second inseminations is elevated, suggests that the Coolidge effect may rather be based on a suppression of mating activity that is strongest in a familiar environment. Similar effects of a novel environment in connection with a Coolidge effect have been found in several vertebrates [30]. In our experiment, this cannot be due to a factor that accumulated in the water, because we used a set-up with continuous water exchange. The explanation should therefore probably be sought in the mucus left on the surface. One explanation could be that a factor in the already-inseminated partner's mucus reduces male mating activity. An alternative explanation could be that the presence of a factor in the mucus from one or more potential partners increases male mating activity. The effect cannot be due to the animals simply using the quantity of mucus as a cue, since the experiment was set up in such a way that the same amount of mucus should have been deposited in both treatments. The latter supports the idea that these animals seem able to distinguish between trails from individuals that they did and did not inseminate before.

Based on the current data we cannot distinguish between whether pond snails can recognise trails from partners that they previously inseminated themselves or trails from individual snails. Although the former seems most parsimonious, in both scenarios either the chemical or textural composition of the mucus could mediate this response to sperm competition. Such a cue in the mucus trail could allow animals to detect the presence of competition. For a very different hermaphrodite, the flatworm *Macrostomum lignano*, Schärer and Ladurner [31] already suggested that a mechanism should be present to differentiate between previously mated and novel partners. *L. stagnalis *does not seem to recognize novel individuals by means of direct contact or water-borne chemical signals, but may rather recognise mucus trails of novel individuals. Chemical cues are known to affect other processes in hermaphrodites. For example, in the polychaete *Ophryotrocha diadema *sex allocation is shifted due to chemical cues [32]. In sea slugs of the genus *Aplysia *a pheromone is released from the egg cordon which attracts potential mates and stimulates them to lay eggs on the same site [33]. Such cues could also be involved in detecting previous partners or even in recognising individuals. That this is a possible function for the mucus, is supported by the ample evidence that gastropod mucus is used for a variety of purposes. Examples include homing [34], locating conspecifics [35], and finding prey [36]. Moreover, the directionality of own trails as well as conspecifics' trails can be detected [37,38].

## Conclusion

Our experiments reveal that great pond snails, *L. stagnalis*, perform more inseminations in larger groups and prefer to inseminate novel over familiar partners. Such higher motivation to copulate when a new partner is encountered is known as the Coolidge effect and has never been demonstrated in hermaphrodites. These hermaphroditic snails thus exhibit prudence in the allocation of their expensive male reserves, which is in line with recent work showing that this species also transfers different numbers of sperm depending on the partner's mating history [39].

## Methods

A total of 264 mature specimens of the pond snail *L. stagnalis *of the same age and with a shell length of 2.80 ± 0.14 cm were obtained from our laboratory culture. Prior to testing they were housed individually in perforated polyethene jars (460 ml) placed in one tank with continuous water exchange. The water was kept at 20°C and the light:dark cycle was 12 h:12 h. During a two-week sexual isolation period each snail was provided with one circular disc of lettuce, with a surface area of 19.6 cm^2^, each day. Snails were individually marked with black nail polish and only used once.

All experiments were performed in glass aquaria (16 × 23.5 × 5 cm) in which glass separations could be placed to create different compartment sizes. During each experiment the water was kept at 20°C and was continuously exchanged. The light:dark cycle was 12 h:12 h. Experiments were recorded via an infra-red camera on a digital hard disk recorder and analysed afterwards by a naïve observer.

To determine the effect of group size on mating frequency, 72 animals were randomly assigned to one of three different group sizes: Pairs, Quartets, and Octets. 24 animals were observed for each group size, hence there were respectively 12, 6, 3 replicates. Because an aquarium could only contain 8 animals, for each group size there were 3 runs that were performed in random order. The water volume per animal was kept constant by placing one (for Quartets) or three (for Pairs) separations in the aquarium. We recorded all mating interactions that occurred during 24 hours.

To compare the effect of a novel partner on male motivation, with the effect of a second presentation of the same partner, a total of 96 pairs of snails were allowed to copulate for an initial 6 hours at the start of the light cycle. These animals were then treated according to either one of two protocols, termed "Dirty" and "Clean". In the "Dirty" protocol, all the snails were briefly lifted out of the aquarium and were returned either with the same partner (Same) or paired with a new partner (from another compartment; New) to their original compartment in the uncleaned aquarium. These pairs were then observed for another 6 hours. In the "Clean" protocol, the only difference in the experimental procedure was that all animals were transferred to a clean aquarium for the second 6 hours of observation.

Evidently, this latter experimental variant excluded mucus trails on the glass as well as other chemicals deposited by the animals. Both variants of the protocol were performed twice and these replicates were done in alternation. Each replicate consisted of six runs with four pairs each, thus resulting in 24 pairs per replicate. Animals were randomly assigned to the treatment groups New or Same. For the data analyses of these experiments we used JMP 5.0.1, and we only included pairs in which both snails had donated sperm during the first 6 hours (thus excluding differences in receptivity of partners).

## Authors' contributions

JMK conceived the experiment and carried it out. JMK analysed the 24 hour recordings of different group sizes. ATM analysed the 12 hour recordings of pairs. Both authors designed the study, performed the statistical analyses, drafted the manuscript, and approved the final version.
